# Improving identification of pulmonary embolism-related out-of-hospital cardiac arrest to optimize thrombolytic therapy during resuscitation

**DOI:** 10.1186/s13054-019-2672-6

**Published:** 2019-12-13

**Authors:** François Javaudin, Jean-Baptiste Lascarrou, Hyacinthe Esquina, Valentine Baert, Hervé Hubert, Brice Leclère, on behalf of GR-RéAC, François Javaudin, Jean-Baptiste Lascarrou, Hyacinthe Esquina, Valentine Baert, Hervé Hubert, Brice Leclère

**Affiliations:** 1 Department of Emergency Medicine, University Hospital of Nantes, Nantes, France; 2 Microbiotas Hosts Antibiotics and bacterial Resistances (MiHAR), University of Nantes, Nantes, France; 3 Medical Intensive Care Unit, University Hospital of Nantes, Nantes, France; 4 Public Health Department EA 2694, Lille University Hospital, University of Lille, Lille, France; 5 Department of Epidemiology and Medical Evaluation, University Hospital of Nantes, Nantes, France

**Keywords:** Out-of-hospital cardiac arrest, Thrombolytic therapy, Pulmonary embolism, Cardiopulmonary resuscitation

Pulmonary embolism (PE) is responsible for ~ 3% of Out-of-Hospital Cardiac Arrest (OHCA) and is associated with unfavorable prognoses [1]. We have recently shown that thrombolysis during resuscitation was associated with a better survival in the event of a proven pulmonary embolism [2]. The challenge is thus to identify, from the beginning of resuscitation, PE-related OHCA in order to deliver the proper treatment to the patient. This issue is highlighted by the premature stoppage of the TROICA trial [1], which showed no benefit of using thrombolysis in medical cardiac arrests. Our aim was to identify the factors associated with PE-related OHCA.

We selected adults from the French National OHCA Registry, admitted to the hospital. This registry and the OHCA management by a mobile medical team have been previously described [3]. The present study was approved by the French Advisory Committee on Information Processing in Health Research. It was approved as a medical assessment registry without a requirement for patient consent.

We assessed characteristics associated with PE-related OHCA with a univariable analysis (*χ*^2^ test, Fisher’s exact test or Student’s *t* test). A multivariate logistic regression model was then developed to identify the factors associated with PE with a *P* value < 0.20. Statistical analyses were performed using R software v3.6.1.

From July 2011 to March 2018, 14,253 patients were admitted to the hospital. We excluded OHCAs whose cause was obvious from the beginning of the resuscitation (*n* = 2341) or where data was incomplete (*n* = 1150). The final analysis included 10,402 subjects. Two hundred sixty subjects (2%) were diagnosed with PE upon hospital admission by computed tomography pulmonary angiography (CTPA) or echocardiogram. The results of the univariate and multivariate analyses are presented in Table 1. The prevalence of PE was 22% among the population with nonshockable rhythm and history of thromboembolism. These two factors had a sensitivity of 22% (95% CI [10–39]), a specificity of 98% (95% CI [97–98]) to detect PE. When an age limitation of < 50 years was added to these two factors, the probability of PE was 44% (Fig. 1).

**Table 1:** Factors associated with OHCA caused by PE

	PE-related OHCA (*n* = 260)	Other etiologies (*n* = 10,142)	Chi^2^ Pearson *p*	Multivariate logistic regression adjusted odds ratio [95% CI]	*p*
Female, *n* (%)	139 (53.5)	3204 (31.6)	< 0.001	2.0 [1.5–2.5]	< 0.001
Age < 50 y, *n* (%)	67 (25.8)	2101 (20.7)	0.048	1.5 [1.1–2.0]	0.01
Absence of known heart disease, *n* (%)	170 (65.4)	5790 (57.1)	0.008	1.3 [1.0–1.7]	0.05
History of respiratory disease, *n* (%)	35 (13.4)	1350 (13.3)	0.94		
History of diabetes, *n* (%)	34 (13.1)	1407 (13.9)	0.71		
Absence of known comorbidities, *n* (%)	43 (16.5)	1431 (14.1)	0.27		
History of thromboembolism, *n* (%)	8 (3.1)	41 (0.4)	< 0.001^a^	6.4 [2.7–13.5]	< 0.001
History of cancer, *n* (%)	25 (9.6)	535 (5.3)	0.002	1.6 [1.0–2.3]	0.04
Initial nonshockable, No. (%)	245 (94.2)	5947 (58.6)	< 0.001	10.4 [6.4–18.4]	< 0.001

^a^Fisher’s exact test

*PE* pulmonary embolism, *OHCA* out-of-hospital cardiac arrest

**Fig. 1:**
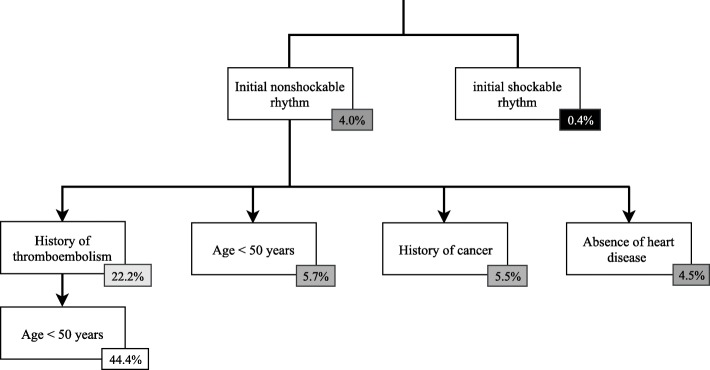
Tree representation of the frequency of pulmonary embolism according to risk factors

In summary, this study found two factors strongly associated with PE: initial nonshockable rhythm and prior thromboembolism. These factors had already been described by Bouguoin et al. [4] as the two major factors associated with the diagnosis of PE-related OHCA. Moreover, in our study, young age was a risk factor for PE, and this population has a lower risk of major bleeding in the case of thrombolysis, as shown in the PEITHO trial (lower risk if ≤ 75 years) [5].

Our study had some limitations such as the lack of completeness of data which may have resulted in the selection of the population not being completely exhaustive. Moreover, the method of confirming PE either by CTPA or echocardiogram was not known for each patient, which may have underestimated the number of PE cases due to the lack of sensitivity for the ultrasound. In addition, autopsy results were not included in the data. Finally, we were unable to include subjects who died on site and were not admitted to hospital because of a lack of confirmation of the etiology of OHCA.

In conclusion, we recommend that for cases of OHCA for which a cause is not obvious, suspect a pulmonary embolism if the initial rhythm is nonshockable and there is a medical history of thromboembolism. In accordance with the guidelines of the American Heart Association (AHA) [6], these subjects should be treated by thrombolysis during resuscitation, especially when they are young.

## Data Availability

All data that were collected where listed in an anonymous database. The dataset is not available but can be requested from the corresponding author.

## Abbreviations

OHCA: Out-of-hospital cardiac arrest
PE: Pulmonary embolism
RéAC: French National OHCA Registry
95% CI: 95% confidence interval
CTPA: Computed tomography pulmonary angiography
AHA: American Heart Association
